# Complete response to PD-1 blockade following EBV-specific T-cell therapy in metastatic nasopharyngeal carcinoma

**DOI:** 10.1038/s41698-021-00162-7

**Published:** 2021-03-19

**Authors:** Corey Smith, Margaret McGrath, Michelle A. Neller, Katherine K. Matthews, Pauline Crooks, Laetitia Le Texier, Benedict Panizza, Sandro Porceddu, Rajiv Khanna

**Affiliations:** 1 QIMR Berghofer Centre for Immunotherapy and Vaccine Development and Tumour Immunology Laboratory, Department of Immunology, QIMR Berghofer Medical Research Institute, Brisbane, Queensland Australia; 2 Princess Alexandra Hospital, Faculty of Medicine, University of Queensland, Brisbane, Australia

**Keywords:** Cancer immunotherapy, Tumour virus infections

## Abstract

Nasopharyngeal carcinoma (NPC) is an Epstein–Barr virus (EBV)-associated heterogeneous disease and is characterized by peritumoral immune infiltrate. Adoptive T-cell therapy (ACT) has emerged as a potential therapeutic strategy for NPC. However, the tumor microenvironment remains a major roadblock for the successful implementation of ACT in clinical settings. Expression of checkpoint molecules by malignant cells can inhibit the effector function of adoptively transferred EBV-specific T cells. Here we present a novel case report of a patient with metastatic NPC who was successfully treated with a combination of EBV-specific ACT and programmed cell death-1 blockade therapy. Following combination immunotherapy, the patient showed complete resolution of metastatic disease with no evidence of disease relapse for 22 months. Follow-up immunological analysis revealed dramatic restructuring of the global T-cell repertoire that was coincident with the clinical response. This case report provides an important platform for translating these findings to a larger cohort of NPC patients.

## Introduction

Nasopharyngeal carcinoma (NPC) remains a significant burden in regions of South-East Asia, with incidences as high as 20 per 100,000 in regions of Southern China^1^. Rates of diagnosis worldwide exceed 70,000 cases annually^2^. While modern radiotherapy and concurrent platinum-based chemotherapy offer high locoregional control rates, ranging between 60% and 80% for locally advanced disease, outcomes for recurrent and distant metastatic disease is poor, with 5-year survival rates less than 50%^3^. Recent studies using checkpoint inhibitors have demonstrated some efficacy as monotherapy in patients with recurrent or metastatic NPC, with overall response rates of 20–34% and evidence of enhanced efficacy in combination with chemotherapy^4–7^. However, few patients have displayed durable long-term responses following checkpoint inhibition.

Endemic NPC is universally associated with the presence of Epstein–Barr virus (EBV)^8,9^, which establishes a latency program within the malignant cells that is characterized by a restricted gene expression profile^10^. Although not definitively proven, EBV likely contributes to the oncogenic potential of NPC cells^11^. Following on from successes in the treatment of EBV-associated post-transplant lymphoma, EBV-specific adoptive T-cell therapy (ACT) is emerging as a potential therapeutic option for NPC^12^. We recently reported on an ACT approach for patients with metastatic NPC using an adenoviral vector encoding EBV epitopes, AdE1-LMPpoly, to generate autologous NPC-targeted EBV-specific T cells^13^. We have since initiated a phase I/II study to assess this approach as an adjunct to chemotherapy. In this case study, we described the outcome of a patient recruited to our ACT study, who received anti-PD-1 (nivolumab) checkpoint blockade therapy on compassionate grounds immediately following the completion of ACT. The patient, whose primary tumor expressed high levels of PDL-1, displayed rapid and complete disease resolution following nivolumab administration and has since shown no evidence of disease recurrence by PET/CT for 22 months following complete response.

## Results

### Case report

**Fig. 1:**
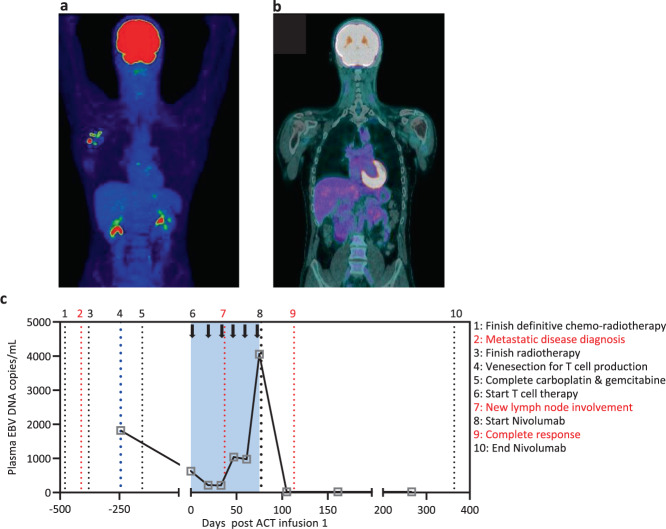
Tumor burden before and after ACT and checkpoint inhibition. **a** PET/CT demonstrating right axilla nodal metastases. **b** Re-staging PET/CT from October 2018 showing complete resolution of disease in the right axilla. **c** Plasma EBV DNA load was measured using standard quantitative PCR designed to target the BamHI-W region. Patient treatment and clinical diagnosis relative to the administration of EBV-specific ACT and nivolumab are overlaid. The period of ACT is represented by the blue box.

A 42-year-old male patient had an initial diagnosis of T4N2M0 (American Joint Commission on Cancer 7^th^ Edition) poorly differentiated NPC that was EBER positive. Disease extended to involve the clivus and right cavernous sinus. The patient was offered definitive chemo-radiotherapy. They were prescribed 70 Gy to gross disease, 63 Gy to the intermediate-risk regions, and 56 Gy to the elective nodal regions in 35 fractions over 7 weeks using volumetric modulated arc therapy along with concurrent high-dose cisplatin in weeks 1 and 4 and 7 (100 mg/m^2^). Week 7 chemotherapy was omitted due to hematologic toxicity. Treatment was completed in February 2017. In May 2017, the patient was diagnosed with metastatic disease involving the right inferior scapular wing and received stereotactic body radiation therapy, 24 Gy in two fractions one week part. In June 2017, the patient proceeded to receive four cycles of palliative carboplatin/gemcitabine chemotherapy, with a 25% reduction in carboplatin and 15% reduction in gemcitabine from cycle 2, which was completed in January 2018. Prior to the commencement of palliative chemotherapy, the patient consented to participate in a prospective phase I/II study (P1486) using EBV-specific ACT for advanced metastatic NPC following standard-of-care treatment. This study is registered under the Australian New Zealand Clinical Trials Registry (ACTRN12613000866707) and has been approved by the human ethics committees of QIMR Berghofer Medical Research Institute and Princess Alexandra Hospital. Venesection was performed and EBV-specific T cells were expanded from peripheral blood mononuclear cells (PBMC) as previously described^14^. From a starting number of 6 × 10^7^ PBMC, 8 × 10^8^ T cells were generated in 14 days, which were cryopreserved at 4 × 10^7^ cells/vial. The cell therapy product consisted of 22% CD3^+^CD8^+^ T cells, of which 21.6% showed EBV-specific reactivity. Therefore, 4.8% of the total cells in the cell therapy product were EBV-specific CD3 + CD8 + T cells The remaining cells in the product were predominantly CD3^+^CD4^+^ T cells (68%). Assessment of EBV-specific reactivity using an overlapping pool of peptides from EBNA1 revealed no response by IFN-γ intracellular cytokine analysis in the CD3^+^CD4^+^ T cell compartment. Prior to the commencement of ACT, the patient showed a reduction in activity in lymph nodes in March 2018. ACT commenced in June 2018 and the patient received six doses of 4 × 10^7^ T cells containing 1.9 × 10^6^ EBV-specific T cells per dose at fortnightly intervals. While the first scan following commencement of ACT displayed a mixed response, the subsequent PET scan in August 2018 showed three new FDG-avid lymph nodes within the right axilla, in between the scapular radiation region and the prior neck irradiation (Fig. 1a). Soon after completing ACT in September 2018, the patient was offered programmed cell death-1 (PD-1) blockade therapy (nivolumab, Bristol-Myers Squibb) on compassionate grounds. The patient was treated with nivolumab at 240 mg per cycle (14 days) from September 2018 until June 2019 (21 cycles). Interestingly, a PET scan in October 2018 (after completion of four cycles of nivolumab) revealed complete resolution of active disease (Fig. 1b). Subsequent scans in February, May, September and December 2019, and March 2020 showed no evidence of disease relapse. Consistent with this response to PD-1 blockade following ACT, immunohistochemical analysis of the primary tumor revealed high expression of PD-L1, with a tumor proportions score of 80%, which represents the proportion of tumor cells expressing PD-L1.

### Virological and immunological monitoring during immunotherapy

At the time of venesection for ACT manufacture and prior to the commencement of chemotherapy, the patient had a detectable EBV DNA load in plasma (Fig. 1c). This is characteristic of the presence of residual tumor burden in EBV-associated NPC^15,16^. The EBV DNA load was reduced around threefold following chemotherapy. After the administration of the first cycle of ACT, the EBV DNA load reduced another threefold; however, during the course of the ACT infusions, the EBV DNA load increased, which was coincident with disease activity in lymph nodes. Prior to the final ACT infusion, the EBV DNA load peaked at 4055 copies/mL. Within 3 weeks of the commencement of nivolumab therapy, EBV DNA was undetectable in the plasma, which was coincident with a diagnosis of complete clinical response by PET (Fig. 1b). EBV DNA remained undetectable more than 250 days after the completion of combination immunotherapy based on autologous EBV-specific T cells and nivolumab and the patient has shown no signs of disease recurrence.

**Fig. 2:**
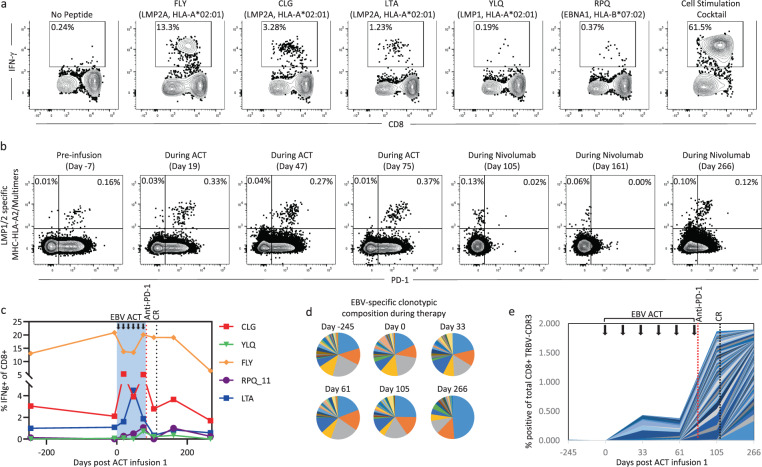
EBV-specific immunological monitoring following immunotherapy. **a** The specificity of T cells generated for cellular therapy was assessed using a standard intracellular cytokine assay following recall with HLA-matched peptide epitopes. Flow cytometry plots show the frequency of CD8^+^ T cells specific for six different EBV-encoded peptides or a positive control cell stimulation cocktail (eBioscience). **b** PBMC isolated during the course of treatment were assessed for PD-1 expression and the presence of EBV-specific T cells using a pool of HLA-A*02:01-restricted MHC dextramers. Plots show the proportion of MHC multimer-specific CD8^+^ T cells that co-express PD-1. **c** PBMC isolated during the course of treatment were stimulated for 2 weeks using an adenoviral vector encoding EBNA1 and multiple CD8^+^ T cell epitopes from LMP1 and LMP2 (AdE1-LMPpoly), then recalled with HLA-matched peptides. Data represent the proportion of IFN-γ producing CD8^+^ T cells in response to each peptide. **d** TRBV-CDR3 deep sequencing analysis was performed on two sets of samples: 1. CD8^+^ T cells sorted from PBMC isolated during the course of treatment, and 2. MHC multimer^+^ CD8^+^ T cells sorted from the ACT product. Pie charts show the proportions of MHC multimer^+^ EBV-specific TRBV sequences from the ACT product that were also detected in the PBMC during the course of treatment. Each wedge represents an individual clonotype and identical clonotypes in each pie chart are represented by the same color. Only clonotypes that were present in the MHC multimer^+^ population from the ACT product are included. **e** The chart shows the frequency of novel clonotypes in the total CD8^+^ T cell population that significantly expanded after the commencement of ACT. Each color represents an individual clonotype. Only clonotypes that significantly expanded after the commencement of ACT are included.

To assess the impact of immunotherapy on EBV-specific T-cell immunity in the patient, we analyzed the ACT product using EBV-specific major histocompatibility complex (MHC) multimers and peptide-specific intracellular cytokine analysis. The ACT product generated for this patient predominantly recognized the HLA-A*02:01-restricted epitopes FLYALALLL (FLY), CLGGLLTMV (CLG), and LTAGFLIFL (LTA) (Fig. 2a). Some reactivity was also detected against the HLA-B*07:02-restricted epitope RPQKRPSCIGC (RPQ), while no response was detectable against other HLA-matched EBV epitopes, including the HLA-A*02:01 restricted epitope YLQQNWWTL (YLQ). Longitudinal MHC multimer analysis using a pool of EBV-specific HLA-A*02:01 multimers indicated that the administration of the ACT product was associated with a change in the proportion of EBV-specific CD8^+^ T cells in PBMC from 0.16% to 0.33% (Fig. 2b). This change in proportion was maintained during the course of ACT. We also noted that the majority of MHC multimer-specific T cells assessed during the course of ACT expressed PD-1. Interestingly, the increased proportion of EBV-specific T cells was associated with changes in the epitope-specific hierarchy of the T-cell response against EBV. During the course of ACT, we saw an increase in the frequency of responses to CLG, LTA, RPH, and YLQ (Fig. 2c). Following the administration of nivolumab, the proportion of EBV-specific T cells in PBMC decreased (Fig. 2b, c). This reduction in T-cell frequency was associated with reduced detection of PD-1 on the surface; however, this may have been due to nivolumab-mediated blocking of the anti-PD-1 antibody used in the analysis.

We next performed T-cell receptor beta variable gene (TRBV) deep sequencing analysis of this patient’s peripheral T-cell repertoire to assess the impact of ACT and checkpoint inhibition. From the ACT product, we sorted EBV-specific MHC multimer^+^ T cells to assess the TRBV repertoire of adoptively transferred antigen-specific T cells. The total CD8^+^ T-cell population was sorted from PBMC isolated during the course of treatment, to track both EBV-specific T cells and changes in the global T-cell repertoire. Deep sequencing analysis was performed using Adaptive Biotechnologies’ ImmunoSeq platform as described previously^17^. Using PBMC, we then assessed the CD8^+^ T cell population over time for clonotypes that were identified as being enriched in the EBV-specific MHC multimer^+^ population. These data are represented as the proportion of the total EBV-specific MHC multimer^+^ population at each time point (Fig. 2d). While clonotypic immunodominance was maintained throughout the course of treatment, with the immunodominant clonotypes remaining dominant at each time point, we saw an increase in the proportion of some subdominant clonotypes during treatment, which could be attributable to either the transferred T cells or the endogenous EBV-specific population. At day 266 after commencement of treatment further changes in clonotypic composition were evident. These were characterized by the contraction of subdominant clonotypes and the emergence of a clearly dominant clonotype that comprised half of the remaining population. These changes were coincident with the contraction seen in the MHC multimer analysis. We also noted the emergence of other novel clonotypes during immunotherapy that were not identified in our EBV-specific analysis (Fig. 2e). This was most evident following nivolumab treatment, with up to 2% of the CD8^+^ T-cell repertoire within the PBMC composed of new clonotypes at day 266 that were not present prior to the commencement of ACT. The emergence of these novel clonotypes may be indicative of epitope spreading to other tumor-associated antigens following the combined immunotherapy approach^18,19^, or represent the expansion of other tumor-specific or EBV-specific clonotypes that were not detected in the cell therapy product.

## Discussion

The observations in the current case report demonstrate the potential impact the combination of EBV-specific ACT followed by checkpoint blockade treatment could have upon patients with advanced EBV-associated malignancies. What remains unclear from the current analysis is the impact of each immunotherapy approach on outcome. Previous observations have shown complete responses following blockade of the PD-1/PD-L1 axis in NPC patients and objective responses have been associated with PD-L1 positive tumors. However, the phase II multicenter international study of nivolumab by Ma and colleagues demonstrated only a single complete response in a study cohort of 44 patients with recurrent disease^5^. Similarly, no NPC patients in the KEYNOTE-028 study demonstrated a complete response to pembrolizumab^4^. This is despite the reported high expression of PD-L1 on NPC tumor cells, with 41 of 44 evaluable patients in KEYNOTE-028 positive for PD-L1. These observations suggest that whilst PD-1/PD-L1 blockade can induce good clinical response rates, it is usually insufficient for complete tumor clearance. Similarly, observations with EBV-specific ACT in NPC patients by our group and others have rarely shown complete clinical responses in patients with advanced disease. Therefore, whilst it is conceivable that checkpoint blockade was sufficient to induce a complete response, it is also plausible that the combined treatment promoted the rapid and profound disease resolution that occurred in this patient.

While it remains to be determined how translatable the findings from this current case will be in a larger cohort of NPC patients, current options for patients with relapsed metastatic NPC are limited. Recent analyses have shown the benefit of adjuvant chemotherapy for improved survival in locally advanced NPC^20^, including reduced incidence of recurrence; however, its efficacy in recurrent metastatic disease has not been formally assessed. Although improved progression-free survival in metastatic disease has been formally demonstrated using gemcitabine plus cisplatin, less than 10% of patients experience a complete response^21^. Augmentation of this response using the anti-PD-1 antibody, camrelizumab, was reported in an early clinical study^6^. However, consistent with previous observations with checkpoint monotherapy, few patients displayed a complete response (2%), further emphasizing the difficulty in generating durable complete responses in patients with metastatic NPC. Data presented in this case report provides an important platform to explore combination therapy based on anti-PD-1 and anti-EBV T cell therapy to further improve the overall survival of NPC patients with recurrent metastatic disease.

## Methods

### T-cell therapy

Following written informed consent (as outlined in the study protocol) venesection was performed on the patient prior to commencement of chemo/radiotherapy. EBV-specific T cells were generated using the AdE1-LMPpoly vector as previously described^14^. The patient received six intravenous infusions of in vitro-expanded T cells at a dose of 4 × 10^7^ T cells (2 × 10^7^ cells/m^2^). The patient was monitored for safety, disease progression, and immune reconstitution for 9 months following the completion of ACT.

### Intracellular cytokine assay

The ACT product or laboratory-cultured T cells were stimulated with a custom EBV peptide pool or individual HLA-matched epitopes, then cultured for 4 hours in the presence of GolgiPlug and GolgiStop (BD Bioscience, New Jersey, USA). Cells were washed and stained with anti-CD8-PerCP-Cy5.5 (clone RPA-T8, eBioscience, San Diego, USA) and anti-CD4-Pacific Blue (clones RPA-T4, BD Biosciences), fixed and permeabilized with Cytofix/Cytoperm (BD Biosciences), washed again and stained with anti-IFN-γ-AF700 (clone B27, BD Biosciences). Cells were washed, then resuspended in PBS and acquired using a BD LSRFortessa with FACSDiva software (BD Biosciences). Post-acquisition analysis was performed using FlowJo software (BD Biosciences). Representative gating analysis is provided in the Supplementary Figure.

### MHC multimer analysis

PBMC were incubated with APC-conjugated HLA-A*02:01 MHC dextramers (FLYALALLL, CLGGLLTMV, YLLEMLWRL, and YLQQNWWTL; Immudex, Copenhagen, Denmark), followed by anti-CD8-SB780 (clone RPA-T8), anti-CD4-AF700 (clone RPA-T4, BD Biosciences), anti-CD95-PE-Cy7 (clone CX2, Biolegend, San Diego, USA), anti-PD-1-BV421 (clone EH12.1, BD Biosciences) and LIVE/DEAD fixable near-IR dead cell stain (ThermoFischer, Waltham, USA). Cells were washed, then resuspended in PBS and acquired using a BD LSRFortessa with FACSDiva software. Post-acquisition analysis was performed using FlowJo software. Representative gating analysis is provided in the Supplementary Figure.

### Isolation and deep sequencing of T cells

To isolate EBV-specific T cells, the ACT product was incubated with APC-conjugated HLA-A*02:01 MHC dextramer FLYALALLL and APC-conjugated HLA-A*02:01 MHC dextramer CLGGLLTMV, followed by anti-CD8-PerCP-Cy5.5 (clone RPA-T8, eBioscience), anti-CD4-FITC (clone RPA-T4, BD Biosciences) and LIVE/DEAD fixable near-IR dead cell stain. MHC dextramer^+^CD8^+^CD4^-^ viable lymphocytes (31,000) were sorted using a BD FACSAria III. To assess changes in the CD8^+^ T cell TRBV landscape following immunotherapy, a minimum of 500,000 CD8^+^CD4^-^ T cells (range 505,126 to 608,470) were sorted from PBMC. DNA was isolated from the sorted cells using the Qiagen DNeasy Kit (Qiagen, Hilden, Germany). The isolated DNA was then sent to Adaptive Biotechnologies® (Seattle, WA, USA) for TRBV deep sequencing analysis using the immunoSEQ platform. Data analysis was performed using the immunoSEQ Analyzer platform. The total number of productive reads was 8,037 for MHC-dextramer^+^ cells, and ranged from 64,192 to 146,379 for CD8^+^CD4^−^ T cells from PBMC.

### Reporting summary

Further information on research design is available in the Nature Research Reporting Summary linked to this article.

## Supplementary information

Supplementary Figure

REPORTING SUMMARY

## Data Availability

The data generated and analyzed during this study are described in the following data record: 10.6084/m9.figshare.13663634^22^. The T-cell receptor beta variable gene (TRBV) deep sequencing is available via the Adaptive Biotechnologies immuneACCESS database (https://clients.adaptivebiotech.com/immuneaccess). These data can be accessed via 10.21417/CS2021NJPPO^23^. The tumor progression and remission (jpeg images), plasma EBV DNA (Excel spreadsheet), intracellular cytokine analysis (Excel spreadsheet), and phenotyping data (Excel spreadsheet) are not publicly available for the following reason: data contain information that could compromise research participant privacy. However, the data can be made available upon reasonable request to the corresponding author.

## References

[1] Yu MC, Yuan JM (2002). Epidemiology of nasopharyngeal carcinoma. Semin Cancer Biol..

[2] Ferlay J (2015). Cancer incidence and mortality worldwide: sources, methods and major patterns in GLOBOCAN 2012. Int J. Cancer.

[3] Liu Q, Chen JO, Huang QH, Li YH (2013). Trends in the survival of patients with nasopharyngeal carcinoma between 1976 and 2005 in Sihui, China: a population-based study. Chin. J. Cancer.

[4] Hsu C (2017). Safety and antitumor activity of pembrolizumab in patients with programmed death-ligand 1-positive nasopharyngeal carcinoma: results of the KEYNOTE-028 study. J. Clin. Oncol..

[5] Ma BBY (2018). Antitumor activity of nivolumab in recurrent and metastatic nasopharyngeal carcinoma: An International, Multicenter Study of the Mayo Clinic Phase 2 Consortium (NCI-9742). J. Clin. Oncol..

[6] Fang W (2018). Camrelizumab (SHR-1210) alone or in combination with gemcitabine plus cisplatin for nasopharyngeal carcinoma: results from two single-arm, phase 1 trials. Lancet Oncol..

[7] Masterson L (2020). Immune checkpoint inhibitors in advanced nasopharyngeal carcinoma: beyond an era of chemoradiation?. Int J. Cancer.

[8] Niedobitek G (1991). Epstein-Barr virus and carcinomas: undifferentiated carcinomas but not squamous cell carcinomas of the nasopharynx are regularly associated with the virus. J. Pathol..

[9] Nicholls JM, Agathanggelou A, Fung K, Zeng X, Niedobitek G (1997). The association of squamous cell carcinomas of the nasopharynx with Epstein-Barr virus shows geographical variation reminiscent of Burkitt’s lymphoma. J. Pathol..

[10] Niedobitek G (1992). Expression of Epstein-Barr virus genes and of lymphocyte activation molecules in undifferentiated nasopharyngeal carcinomas. Am. J. Pathol..

[11] Raab-Traub N (2012). Novel mechanisms of EBV-induced oncogenesis. Curr. Opin. Virol..

[12] Smith C, Khanna R (2015). Adoptive therapy for EBV-induced cancers: driving success with post-transplant lymphoproliferative disorder to other EBV-derived tumors. Immunotherapy.

[13] Smith C (2017). Pre-emptive and therapeutic adoptive immunotherapy for nasopharyngeal carcinoma: phenotype and effector function of T cells impact on clinical response. Oncoimmunology.

[14] Smith C (2012). Effective treatment of metastatic forms of Epstein-Barr virus-associated nasopharyngeal carcinoma with a novel adenovirus-based adoptive immunotherapy. Cancer Res..

[15] Leung SF (2014). Plasma Epstein-Barr viral DNA load at midpoint of radiotherapy course predicts outcome in advanced-stage nasopharyngeal carcinoma. Ann. Oncol..

[16] Wang WY (2016). Long-term clinical outcome in nasopharyngeal carcinoma patients with post-radiation persistently detectable plasma EBV DNA. Oncotarget.

[17] Smith C (2019). T cell repertoire remodeling following post-transplant T cell therapy coincides with clinical response. J. Clin. Invest.

[18] Zacharakis N (2018). Immune recognition of somatic mutations leading to complete durable regression in metastatic breast cancer. Nat. Med..

[19] Akyuz N (2017). T-cell diversification reflects antigen selection in the blood of patients on immune checkpoint inhibition and may be exploited as liquid biopsy biomarker. Int J. Cancer.

[20] Ribassin-Majed L (2017). What is the best treatment of locally advanced nasopharyngeal carcinoma? An individual patient data network meta-analysis. J. Clin. Oncol..

[21] Zhang L (2016). Gemcitabine plus cisplatin versus fluorouracil plus cisplatin in recurrent or metastatic nasopharyngeal carcinoma: a multicentre, randomised, open-label, phase 3 trial. Lancet.

[22] Smith, C. e. a. *Metadata record for the manuscript: Complete Response to PD-1 Blockade Following EBV-specific T-cell Therapy in Metastatic Nasopharyngeal Carcinoma. figshare*10.6084/m9.figshare.13663634, (2021). 10.1038/s41698-021-00162-7 PMC7979738 33742086

[23] ImmuneACCESS. 10.21417/ADPT2020COVID, (2021).

