# Supplementary material for: Complete response to PD-1 blockade following EBV-specific T-cell therapy in metastatic nasopharyngeal carcinoma
Source: NPJ Precis Oncol. 2021 Mar 19;5:24. doi: 10.1038/s41698-021-00162-7 (PMC7979738; doi:10.1038/s41698-021-00162-7)
Supplement: Supplementary file 1 — Supplementary Figure [file 41698_2021_162_MOESM1_ESM.pdf]

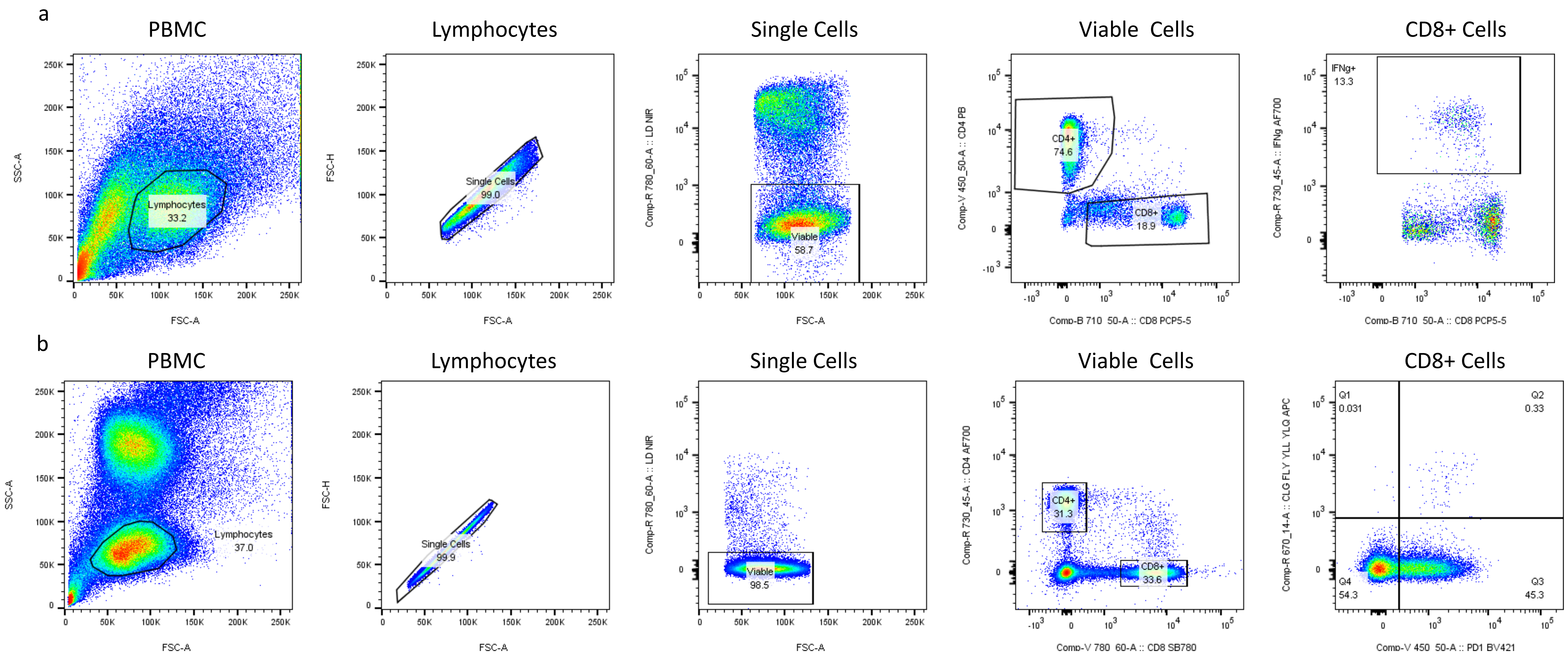


**Supplementary Figure:** Representative Flow Cytometry Staining for intracellular cytokine analysis **(a)** and MHC-multimer analysis **(b)**. T cells (**a**) or PBMC (**b**) were first gated in lymphocytes, followed by double and non-viable cell exclusion. Cells were then gated on CD8+CD4- cells, followed by assessment of either IFN-gamma (**a**) or MHC-Multimer (CLG, FLY, YLL, YLQ) and PD-1 (**b**) in CD8+ T cells.
