# Supplementary material for: Complete response to PD-1 blockade following EBV-specific T-cell therapy in metastatic nasopharyngeal carcinoma
Source: NPJ Precis Oncol. 2021 Mar 19;5:24. doi: 10.1038/s41698-021-00162-7 (PMC7979738; doi:10.1038/s41698-021-00162-7)
Supplement: Supplementary file 2 — REPORTING SUMMARY [file 41698_2021_162_MOESM2_ESM.pdf]

nature research

Corresponding author(s): Professor Rajiv Khanna
Last updated by author(s): YYYY-MM-DD

Reporting Summary

Nature Research wishes to improve the reproducibility of the work that we publish. This form provides structure for consistency and transparency in reporting. For further information on Nature Research policies, see our Editorial Policies and the Editorial Policy Checklist.

Statistics

For all statistical analyses, confirm that the following items are present in the figure legend, table legend, main text, or Methods section.

n/a	Confirmed	
☒	☐	The exact sample size (*n*) for each experimental group/condition, given as a discrete number and unit of measurement
☒	☐	A statement on whether measurements were taken from distinct samples or whether the same sample was measured repeatedly
☒	☐	The statistical test(s) used AND whether they are one- or two-sided<br>*Only common tests should be described solely by name; describe more complex techniques in the Methods section.*
☒	☐	A description of all covariates tested
☒	☐	A description of any assumptions or corrections, such as tests of normality and adjustment for multiple comparisons
☒	☐	A full description of the statistical parameters including central tendency (e.g. means) or other basic estimates (e.g. regression coefficient) AND variation (e.g. standard deviation) or associated estimates of uncertainty (e.g. confidence intervals)
☒	☐	For null hypothesis testing, the test statistic (e.g. *F*, *t*, *r*) with confidence intervals, effect sizes, degrees of freedom and *P* value noted<br>*Give P values as exact values whenever suitable.*
☒	☐	For Bayesian analysis, information on the choice of priors and Markov chain Monte Carlo settings
☒	☐	For hierarchical and complex designs, identification of the appropriate level for tests and full reporting of outcomes
☒	☐	Estimates of effect sizes (e.g. Cohen's *d*, Pearson's *r*), indicating how they were calculated

Our web collection on statistics for biologists contains articles on many of the points above.

Software and code

Policy information about availability of computer code

Data collection: *Provide a description of all commercial, open source and custom code used to collect the data in this study, specifying the version used OR state that no software was used.*

Data analysis: *Provide a description of all commercial, open source and custom code used to analyse the data in this study, specifying the version used OR state that no software was used.*

For manuscripts utilizing custom algorithms or software that are central to the research but not yet described in published literature, software must be made available to editors and reviewers. We strongly encourage code deposition in a community repository (e.g. GitHub). See the Nature Research guidelines for submitting code & software for further information.

Data

Policy information about availability of data

All manuscripts must include a data availability statement. This statement should provide the following information, where applicable:

- Accession codes, unique identifiers, or web links for publicly available datasets
- A list of figures that have associated raw data
- A description of any restrictions on data availability

The datasets generated during and/or analysed during the current study are available from the corresponding author on reasonable request.

Field-specific reporting

Please select the one below that is the best fit for your research. If you are not sure, read the appropriate sections before making your selection.

☒ Life sciences ☐ Behavioural & social sciences ☐ Ecological, evolutionary & environmental sciences

For a reference copy of the document with all sections, see nature.com/documents/nr-reporting-summary-flat.pdf

Life sciences study design

All studies must disclose on these points even when the disclosure is negative.

Sample size	This is a case report and describes results from a single donor
Data exclusions	No data was excluded from the analysis
Replication	This is a single patient case report
Randomization	Not applicable due to report of a single donor
Blinding	Not applicable

Reporting for specific materials, systems and methods

We require information from authors about some types of materials, experimental systems and methods used in many studies. Here, indicate whether each material, system or method listed is relevant to your study. If you are not sure if a list item applies to your research, read the appropriate section before selecting a response.

Materials & experimental systems

n/a	Involved in the study	
☐	☒	Antibodies
☒	☐	Eukaryotic cell lines
☒	☐	Palaeontology and archaeology
☒	☐	Animals and other organisms
☐	☒	Human research participants
☐	☒	Clinical data
☒	☐	Dual use research of concern

Methods

n/a	Involved in the study	
☒	☐	ChIP-seq
☐	☒	Flow cytometry
☒	☐	MRI-based neuroimaging

Antibodies

Antibodies used	Antibodies used in this study: anti-CD8-PerCP-Cy5.5 (clone RPA-T8, eBioscience), anti-CD4-Pacific Blue (clones RPA-T4, BD Biosciences), anti-IFN-g-Alexa Fluor 700 (clone B27, BD Biosciences), anti-CD8-SB780 (clone RPA-T8, eBioscience), anti-CD4-AF700 (clone RPA-T4, BD Biosciences) anti-CD95-PECy7 (clone CX2, Biolegend, San Diego, USA), anti-PD-1-BV421 (clone EH12.1, BD Biosciences), anti-CD4-FITC (clone RPA-T4, BD Biosciences, New Jersey, USA.
Validation	All antibodies used in this study have been previously validated in the laboratory. All antibodies are tested and titrated on human PBMC samples as per the manufacturer's recommendations.

Human research participants

Policy information about studies involving human research participants

Population characteristics	The manuscript describes a single case report of a patient.
Recruitment	The patient was recruited as part of a clinical trial and were selected based on eligibility criteria. See ACTRN12613000866707 for criteria. We are no aware of any bias in recruitment
Ethics oversight	QIMR Berghofer Medical Research Institute and Princess Alexandra Hospital.

Note that full information on the approval of the study protocol must also be provided in the manuscript.

Clinical data

Policy information about clinical studies

All manuscripts should comply with the ICMJE guidelines for publication of clinical research and a completed CONSORT checklist must be included with all submissions.

Clinical trial registration	ACTRN12613000866707
Study protocol	The study protocol can share on requested via the corresponding author
Data collection	Data were collected at Princes Alexandra Hospital and QIMR-Berghofer. Data was collected from the middle of 2017 until present day
Outcomes	Although this is a case report, the clinical trial has outcome measures which are described in the approved protocol, including safety and efficacy

Flow Cytometry

Plots

Confirm that:

☒ The axis labels state the marker and fluorochrome used (e.g. CD4-FITC).

☒ The axis scales are clearly visible. Include numbers along axes only for bottom left plot of group (a 'group' is an analysis of identical markers).

☒ All plots are contour plots with outliers or pseudocolor plots.

☒ A numerical value for number of cells or percentage (with statistics) is provided.

Methodology

Sample preparation	All biological material from the patient was prepared from the peripheral blood of the patient at the indicated timepoints. Plasma for EBV DNA load was isolated following centrifugation of whole blood and stored at -70C. Peripheral blood mononuclear cells (PBMC) were prepared from whole blood using a FICOLL-Paque density gradient and cryopreserved in 10% DMSO in liquid nitrogen until required for analysis. PBMC were thawed rapidly from cryopreservation, then washed to remove DMSO and used for flow cytometric or funcitonal analysis
Instrument	Samples were acquired using a BD LSRFortessa with FACSDiva software (BD Biosciences, Franklin Lakes, New Jersey, USA).
Software	Post-acquisition cytokine analysis was performed using FCAP array (BD Biosciences) software.<br>Graphs were compiled using GraphPad Prism. TCR sequencing was performed by Adaptive Biotechnologies and data analysis was performed using the immunoSEQ Analyzer
Cell population abundance	Acquisition for flow cytometry was set to acquire a minimum 50,000 viable lymphocytes for cultured T cells and 100,000 viable cells for PBMC. Antigen-specific cell abundance for ICS and MHC-Multimer analysis ranged from 0.06% to 26% of CD8+ T cells.
Gating strategy	All gating strategies commenced with FSC-A vs SSC-A to define lymphocytes, followed by a single cell gate FSC-H vs FSC-A to exclude doublets. Non-viable cells were then exlcuded using Live-Dead NIR vs FSC-A gate. CD4 vs CD8 gate was next used to separate CD8+ cells (CD8+CD4-) . For ICS analysis cells, IFNg production was then assessed in CD8+ T cells using IFNg vs CD8 gate. MHC-Multimer vs PD-1 on CD8+ T cells was used for frequency and checkpoint analysis

☒ Tick this box to confirm that a figure exemplifying the gating strategy is provided in the Supplementary Information.
